# Quantifying the Ki-67 Heterogeneity Profile in Prostate Cancer

**DOI:** 10.1155/2013/717080

**Published:** 2013-10-03

**Authors:** Shane Mesko, Patrick Kupelian, D. Jeffrey Demanes, Jaoti Huang, Pin-Chieh Wang, Mitchell Kamrava

**Affiliations:** ^1^UC Irvine School of Medicine, 1001 Health Sciences Road, 252 Irvine Hall, Irvine, CA 92697-3950, USA; ^2^UCLA Department of Radiation Oncology, UCLA Health System, 200 UCLA Medical Plaza, Suite B265, Los Angeles, CA 90095-6951, USA; ^3^Jonsson Comprehensive Cancer Center, 8-684 Factor Building, Box 951781, Los Angeles, CA 90095-1732, USA; ^4^Department of Pathology, UCLA Health Systems, 10833 Le Conte Avenue, CHS 14-112, Los Angeles, CA 90095-1732, USA

## Abstract

*Background:* Ki-67 is a robust predictive/prognostic marker in prostate cancer; however, tumor heterogeneity in prostate biopsy samples is not well studied. *Methods:* Using an MRI/US fusion device, biopsy cores were obtained systematically and by targeting when indicated by MRI. Prostate cores containing cancer from 77 consecutive men were analyzed. The highest Ki-67 was used to determine interprostatic variation. Ki-67 range (highest minus lowest) was used to determine intraprostatic and intralesion variation. Apparent diffusion coefficient (ADC) values were evaluated in relation to Ki-67. *Results:* Interprostatic Ki-67 mean ± standard deviation (SD) values for NCCN low (L), intermediate (I), and high (H) risk patients were 5.1 ± 3.8%, 7.4 ± 6.8%, and 12.0 ± 12.4% (ANOVA *P* = 0.013). Intraprostatic mean ± SD Ki-67 ranges in L, I, and H risk patients were 2.6 ± 3.6%, 5.3 ± 6.8%, and 10.9 ± 12.3% (ANOVA *P* = 0.027). Intralesion mean ± SD Ki-67 ranges in L, I, and H risk patients were 1.1 ± 0.9%, 5.2 ± 7.9%, and 8.1 ± 10.8% (ANOVA *P* = 0.22). ADC values at Ki-67 > and <7.1% were 860 ± 203 and 1036 ± 217, respectively (*P* = 0.0029). *Conclusions:* High risk patients have significantly higher inter- and intraprostatic Ki-67 heterogeneity. This needs to be considered when utilizing Ki-67 clinically.

## 1. Introduction

Progress in multiparametric MRI imaging has improved our ability to visualize specific target lesions within the prostate. Ultrasound/MRI fusion devices allow for targeted biopsies of these specific MRI defined lesions. These advances create an opportunity to evaluate biomarkers from specific target lesions for integration into radiation treatment stratification.

The Ki-67 protein functions as a nuclear antigen that is only expressed in proliferating cells. It is a marker of the growth fraction in malignant tissue [1–3]. It is determined via immunohistochemistry and expressed as a percentage of cells showing activity in a given tissue sample (e.g., Ki-67 of 10% equates to 10% of the cells expressing the antigen). It is a promising biomarker in prostate cancer with independent predictive/prognostic value following radiotherapy [4–6]. A range of percentage cut points has correlated with outcomes but has not been prospectively validated [7–11]. One limitation to integrating biomarkers into clinical practice is being able to account for tumor heterogeneity. Ki-67 heterogeneity has been acknowledged in liver, breast, and several other cancers but has not been well studied in prostate cancer [12–14]. Previous studies have used the highest Ki-67 level found on routine systematic prostate biopsy cores but have not evaluated variation based on MRI defined lesions. Understanding which MRI defined lesions harbor the highest Ki-67 would be helpful in directing targeted biopsies and informing future clinical trial design. In this study we evaluated Ki-67 variation across NCCN risk groups (interprostatic), within individual prostates (intraprostatic), and within MRI-defined individual lesions (intralesion). We also looked at how the highest Ki-67 per patient is related to the most dominant lesion on MRI and whether apparent diffusion coefficient (ADC) values based on diffusion weighted imaging correlate with Ki-67.

## 2. Materials and Methods

This was an IRB approved retrospective study. Charts were reviewed for patients who were referred to the Department of Urology for Artemis (ultrasound/MRI fusion) guided prostate biopsies. All men underwent 3T multiparametric MRI prior to biopsy. Lesions identified on MRI imaging were segmented as regions of interest. The MRI was then fused with ultrasound at the time of the biopsy. Systematic Artemis assisted biopsies were performed first and, when MRI indicated a lesion, targeted biopsies were performed. Targeted biopsies were taken every 3–5 mm through a target.

Patients were stratified by NCCN Risk criteria using pretreatment PSA, T stage, and Gleason score. Pathology reports were reviewed for Gleason score and Ki-67 (%) for each of the positive prostate cancer cores. The highest Ki-67 documented for each patient was used for interprostatic variation. For patients with ≥2 positive biopsies variation within each prostate (intraprostatic) was performed by taking the highest Ki-67 minus the lowest Ki-67. Intralesion analysis was carried out when multiple biopsy cores were taken from one MRI-defined lesion using the same high minus low Ki-67 method used for intraprostatic variation. The index lesion was defined as the one with the maximum tumor diameter as measured on T2 weighted MRI. The ADC values of lesions as determined from diffusion weighted imaging were also examined to determine if there was a correlation with Ki-67.

### 2.1. Ki-Staining Methods

Paraffin-embedded sections were cut at 4 *μ*m thickness and paraffin removed with xylene and rehydrated through graded ethanol. Endogenous peroxidase activity was blocked with 3% hydrogen peroxide in methanol for 10 min. Heat-induced antigen retrieval (HIER) was carried out for all sections in 0.01 M Citrate buffer, pH = 6.00, using a vegetable steamer at 95°C for 25 min. The slides were then stained with mouse monoclonal Ki-67, clone MIB1 (DakoCytomation, M7240), for 45 min at room temperature. The primary antibody was diluted with calcium chloride to 1/100 concentration. The signal was detected using the MACH 2 Mouse HRP Polymer (Biocare Medical, MHRP520). All sections were visualized with the diaminobenzidine reaction and counterstained with hematoxylin.

### 2.2. Statistical Analysis

One-way analysis of variance (ANOVA) was used to evaluate significant differences between the means of the different NCCN Risk groups. Statistical significance was set at a *P*  value < 0.05.

## 3. Results

77 men were identified who had Artemis guided positive prostate biopsies with Ki-67 staining reported. The mean patient age was 67, the mean PSA was 7.7 ng/dL, and all patients had a clinical stage of T2a or lower (see Table 1).

**Figure 1:**
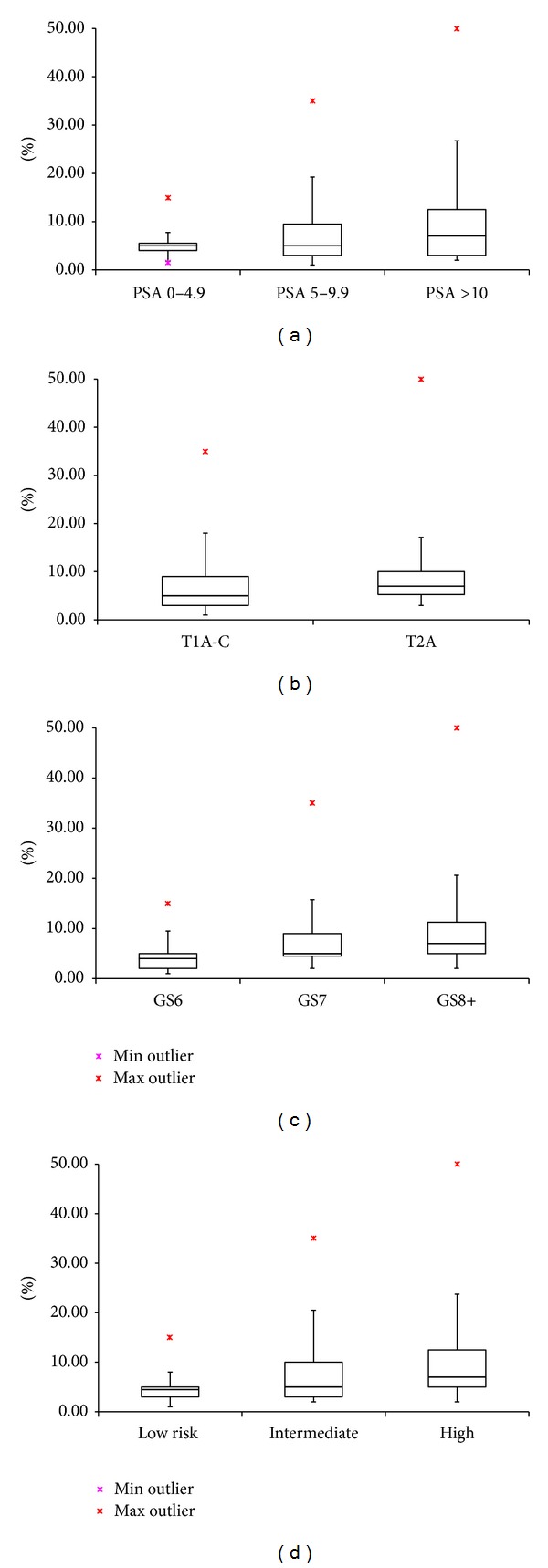
Ki-67% at (a) increasing PSA ranges, (b) clinical T stages, (c) increasing Gleason scores, and (d) NCCN risk groups.

Interprostatic variation showed the Ki-67 ranged from 1 to 50% with an overall mean of 7.4%. Ki-67 was significantly different between NCCN risk groups with mean ± standard deviation (SD) values for low, intermediate, and high risk patients of 5.1% ± 3.8%, 7.4% ± 6.8%, and 12.0% ± 12.4% (ANOVA *P* = 0.013) (Figure 1). It was also significantly different for Gleason scores of 6, 7, and ≥8, with Ki-67 means of 5.0% ± 3.8%, 7.7% ± 7.0%, and 12.0% ± 12.4% (*P* = 0.01, Figure 1). Differences by T stage and PSA were not significant (Figure 1).

**Figure 2:**
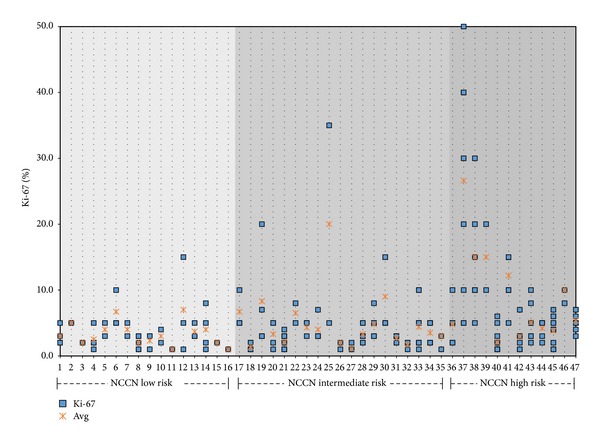
The Ki-67% in each lesion for the 47 patients with ≥2 lesions is demonstrated by each square along with means designated by a red “x.” Patients are stratified by NCCN Risk Group.

Intraprostatic variation was assessed on 47 patients with ≥2 biopsy-positive cores with Ki-67 quantified. Mean ± SD Ki-67 variation (the highest Ki-67 minus the lowest Ki-67) in low, intermediate, and high risk patients was 2.6 ± 3.6%, 5.3 ± 6.8%, and 10.9 ± 12.3% (ANOVA *P* = 0.027).  Figure 2 shows the distribution of Ki-67 values and means for each patient per risk group showing a greater heterogeneity of Ki-67 in higher risk patients.

Intralesion variation was assessed on 38 MP-MRI defined lesions that had ≥2 cores from each lesion with Ki-67 staining. Intralesion mean ± SD Ki-67 variation (the highest Ki-67 minus the lowest Ki-67) in low, intermediate, and high risk patients was 1.1 ± 0.9%, 5.2 ± 7.9%, and 8.1 ± 10.8% (ANOVA *P* = 0.22).

10 patients had 2 or more lesions identified on MP-MRI. The dominant lesion harbored the highest Ki-67 30% of the time (Table 2). The dominant and nondominant lesion contained the same Ki-67 in 30% and in 40% of patients the highest Ki-67 was seen in a nondominant lesion.

Ki-67 cut-off levels of <3.5% and >7.1% were used based on retrospective validation of these values in predicting outcomes following definitive radiation treatment in patients treated on two separate RTOG trials [15, 16]. The mean ± SD ADC in patients with a Ki-67 < 3.5% (*n* = 31) was 1075 ± 205 and in patients with a Ki-67 > 3.5% (*n* = 48) was 940 ± 224 (*P* = 0.0039). For patients with a Ki-67 < 7.1% (*n* = 60) the mean ADC was 1036 ± 217 while patients with values > 7.1% had a mean ADC of 860 ± 203 (*P* = 0.0029).

## 4. Discussion

NCCN risk grouping (clinical T stage, Gleason score, and PSA) is commonly used to determine radiation treatment options. While this risk stratification is clinically helpful, it is also limited in that patients in each risk category are not homogeneous. Integration of biomarkers into existing stratification schemes could help personalize treatment options. Ki-67 is a robust biomarker that has been evaluated in three separate RTOG trials with cut-offs of 3.5%, 6.2%, and 7.1% being independent predictors of outcomes [11, 15–17]. These studies used the highest Ki-67 based on standard systematic prostate biopsy cores. It is possible that this method actually underscores patients, as a standard biopsy may not obtain tissue from areas of the highest risk. To integrate these findings into future clinical studies one needs to consider the impact of tumor heterogeneity so one can be confident that patients are appropriately stratified.

Using information from multiparametric MRI and targeted biopsies we were able to demonstrate a number of things. Overall we found Ki-67 levels are more heterogeneous with increasing NCCN risk group. This observation is consistent with other studies which have shown increased Ki-67 in patients with higher Gleason scores [18, 19]. We also found significant heterogeneity in our intraprostatic and intralesion analysis. Higher risk groups consistently showed a greater degree of variation within each prostate/lesion, but even low risk patients had differences as high as 14% between two locations within a prostate.

Given the variability within the prostate we tried to determine if the index lesion was most likely to harbor the highest Ki-67. We found that this was the case in only 3/10 cases. This suggests that relying on a biopsy only from the index lesion may not be a reliable representation of the highest Ki-67 within the entire gland.

Further complicating things is the high variability within an individual lesion. While 8% variability (the highest Ki-67 minus lowest Ki-67) seems low the cut-offs for Ki-67 levels that stratify patients range between 3.5% and 7.1%. So a difference of 8% is actually very meaningful. The concept of tumor heterogeneity is certainly not novel but this study emphasizes the importance of not relying too heavily on a single core. A more representative picture of the tumor, at least with respect to Ki-67, is better achieved with multiple cores taken from a single lesion. It is beyond the scope of this paper to provide an answer to how many cores are needed to accurately depict the totality of tumor heterogeneity.

Given the complexities with accurately portraying tumor heterogeneity based off of biopsy samples we asked whether the average ADC value correlates with Ki-67 values. This would be meaningful because it would be much simpler to use an ADC value generated from a computer to stratify a patient rather than doing multiple targeted biopsies. We found that there was a significant correlation between ADC values within clinically relevant Ki-67 groupings (i.e., <3.5%, 3.5–7.1%, and >7.1%). There is overlap between these values but their means are significantly different from one another. While this is an interesting correlation, validation on a larger cohort is needed and ultimately prospective data is needed to determine if ADC values can independently predict outcomes in prostate cancer.

## 5. Conclusions

This study provides the first evidence of the magnitude of tumor heterogeneity of the most well studied tumor biomarker in radiation therapy for prostate cancer. Integration of Ki-67 into future risk stratification schemes for clinical trials needs to incorporate issues related to tumor heterogeneity in order to accurately stratify patients.

## Figures and Tables

**Table 1:** Patient characteristics.

	Total	NCCN low	NCCN inter	NCCN high
Number of patients	77	31	30	16
Mean age (years)	66.8	65.7	65.8	70.6
Age range	44–82	44–76	51–82	58–82
Mean PSA (ng/dL)	7.7	4.9	7.22	14.1
PSA range	0.51–36.2	0.51–9.7	0.8–15	2.3–36.2
T1A-C	63 (82%)	30 (97%)	28 (93%)	5 (31%)
T2A	14 (18%)	1 (3%)	2 (7%)	11 (69%)
T2B+	0	0	0	0
Gleason 6	34 (44%)	31 (100%)	3 (10%)	0
Gleason 7	27 (35%)	0	27 (90%)	0
Gleason 8–10	16 (21%)	0	0	16 (100%)
Biopsy-positive cores with Ki-67 stain	268	66	105	97
Mean cores per patient	3.48	2.13	3.50	6.06
Intraprostatic: patients with ≥2 Ki-67 values	47	16	19	12
Intralesion: lesions with ≥2 MRI targeted cores	38	7	15	16

**Table 2:** Comparison of Ki-67% in dominant and nondominant lesions, stratified by NCCN risk group.

	Pt #	Dominant lesion			Nondominant			High Ki-67 in dominant lesion
		MRI lesion (cm)	Ki-67 (%)	GS	MRI lesion (cm)	Ki-67 (%)	GS	
High	7	2.3	7	4 + 5	1.9	7	4 + 4	Yes*
	11	1.6	8	3 + 4	0.9	10	3 + 5	No
	54	3.1	5	5 + 4	1.3	2	5 + 4	Yes
	73	1.9	20	4 + 5	0.8	15	3 + 5	Yes
	74	1.1	50	4 + 5	0.5	5	3 + 4	Yes

Inter	14	1.3	5	3 + 4	0.9	10	3 + 4	No
	27	1	1	3 + 3	0.7	1	3 + 3	Yes*
	58	2.2	1	3 + 3	2	5	3 + 4	No

Low	20	1.3	1	3 + 3	0.8	1	3 + 3	Yes*
	77	1.1	3	3 + 3	0.9	5	3 + 3	No

*High Ki-67 in nondominant lesion also.
